# A Textile Sleeve for Monitoring Oxygen Saturation Using Multichannel Optical Fibre Photoplethysmography

**DOI:** 10.3390/s20226568

**Published:** 2020-11-17

**Authors:** Hattan K. Ballaji, Ricardo Correia, Serhiy Korposh, Barrie R. Hayes-Gill, Francisco U. Hernandez, Byron Salisbury, Stephen P. Morgan

**Affiliations:** 1 Optics and Photonics Group, Faculty of Engineering, University of Nottingham, Nottingham NG7 2RD, UK; hattan.ballaji@nottingham.ac.uk (H.K.B.); Ricardo.GoncalvesCorreia@nottingham.ac.uk (R.C.); S.Korposh@nottingham.ac.uk (S.K.); barrie.hayes-gill@nottingham.ac.uk (B.R.H.-G.); 2 Computer Engineering Department, College of Computers and Information System, Umm Al-Qura University, Makkah 24231, Saudi Arabia; 3 Footfalls and Heartbeats (UK) Ltd., Nottingham NG7 1FW, UK; ulises@footfallsandheartbeats.com (F.U.H.); byron@footfallsandheartbeats.com (B.S.)

**Keywords:** wearable technology, textile, plastic optical fibre, photoplethysmography, oxygen saturation, multichannel

## Abstract

Textile-based systems are an attractive prospect for wearable technology as they can provide monitoring of key physiological parameters in a comfortable and unobtrusive form. A novel system based on multichannel optical fibre sensor probes integrated into a textile sleeve is described. The system measures the photoplethysmogram (PPG) at two wavelengths (660 and 830 nm), which is then used to calculate oxygen saturation (SpO_2_). In order to achieve reliable measurement without adjusting the position of the garment, four plastic optical fibre (POF) probes are utilised to increase the likelihood that a high-quality PPG is obtained due to at least one of the probes being positioned over a blood vessel. Each probe transmits and receives light into the skin to measure the PPG and SpO_2_. All POFs are integrated in a stretchable textile sleeve with a circumference of 15 cm to keep the sensor in contact with the subject’s wrist and to minimise motion artefacts. Tests on healthy volunteers show that the multichannel PPG sensor faithfully provides an SpO_2_ reading in at least one of the four sensor channels in all cases with no need for adjusting the position of the sleeve. This could not be achieved using a single sensor alone. The multichannel sensor is used to monitor the SpO_2_ of 10 participants with an average wrist circumference of 16.0 ± 0.6 cm. Comparing the developed sensor’s SpO_2_ readings to a reference commercial oximeter (reflectance Masimo Radical-7) illustrates that the mean difference between the two sensors’ readings is −0.03%, the upper limit of agreement (LOA) is 0.52% and the lower LOA is −0.58%. This multichannel sensor has the potential to achieve reliable, unobtrusive and comfortable textile-based monitoring of both heart rate and SpO_2_ during everyday life.

## 1. Introduction

Wearable and smart textile technology for health monitoring is an area of great interest for health and wellbeing. New devices have been developed using different sensing technology with the aim of providing reliable unobtrusive monitoring [1,2,3,4]. These devices use discrete sensors integrated into textiles and there are challenges with comfort of users for long-term health monitoring. An alternative approach to comfortable, unobtrusive physiological monitoring is to integrate optical fibres into textiles [5,6,7,8,9] where heart rate and oxygen saturation monitoring have been demonstrated. The advantages of using optical fibres are that they are a low-cost, lightweight, flexible, versatile and robust platform.

Photoplethysmography (PPG) technology is used extensively in optical sensors that are found in medical and fitness monitoring devices. The PPG technique can be implemented in transmission and reflection modes and monitors blood volume changes. Heart rate (HR) is obtained using one wavelength and oxygen saturation (SpO_2_) using two wavelengths as the absorption spectra for oxyhaemoglobin (HbO_2_) and deoxyhaemoglobin (Hb) in blood are different [10,11]. Red and infrared (IR) wavelengths are typically combined to calculate an R value [12,13]:

(1) R= IAC,RED IDC, RED IAC,IR IDC,IR

I_AC_ and I_DC_ for red and IR are the AC and DC light intensities at each wavelength. This “ratio of ratios” R helps to compensate for changes in intensity due to light source fluctuations or fibre bending. According to ISO 80601-2-61:2011, to obtain accurately the SpO_2_ for a specific device requires calibration by taking blood samples (SaO_2_) over a range of values under controlled laboratory conditions [14]. It is found that the SaO_2_ is inversely related to R and hence a calibration curve can be generated. However, a frequently used approximate relationship between R and SpO_2_ that is useful for prototype development is provided in [12,15] as:

(2) $$\%\;\mathrm{Sp}O_{2}=\;110\;-\;25\;R.$$

The light illuminating the tissue and subsequently detected depends on: the light sources and detectors, their separation and number, their position with respect to blood vessels, the relative motion between them and the tissue surface. Signal loss due to the position of the probe can therefore be a problem in photoplethysmography, particularly in life-preserving applications [16,17].

Multichannel PPG sensors that contain multiple sources and detectors have been investigated to overcome problems associated with signal loss. For example, one such sensor detects the reflective PPG from the ear using three photodiodes (PDs) and two IR light-emitting diodes (LEDs) [18]. These PDs and LEDs were used to investigate which channel provided the most reliable PPG signal. The sensor was able to detect the PPG during exercise that would not have been achieved by a single channel sensor. Another similar study built four IR LEDs alongside four PDs, with the configuration based on a wristwatch [19]. This design measured the signal from the radial and ulnar arteries and overcomes the inconvenience of wearing a finger probe during daily life. A circular configuration in which one PD was fixed at the centre with 6 LEDs surrounding it was used to measure the PPG from the forehead [20]. The PPG signals that were acquired simultaneously from independent channels in the sensor were affected differently by artefacts. This forehead sensor used automatic adjudication based on computational algorithms to select the most reliable signal, therefore, helping to minimise measurement error. Alzahrani et al. [21] introduced a multichannel optoelectronic system in order to reduce motion artefacts during everyday activities and exercise. The sensor included one PD in the centre, around which are multiwavelength LEDs arranged in a cross-pattern and multiwavelength LEDs fixed around this. Back-scattered light from multiple wavelengths helps to withstand displacement and misalignment caused by movement. All these multichannel PPG sensor configurations are based on optoelectronic components positioned close to the skin surface.

Greater user comfort can be achieved using optical fibres integrated into textiles, often termed as “photonic textiles” [5,6,7,22,23]. This paper describes the first implementation of a textile-based multichannel SpO_2_ measurement system and an investigation in a human volunteer study. It should be noted that there are several examples of using optical fibres for pulse oximetry [22,24,25] but, these are not integrated into textiles nor use a multichannel approach. A sleeve has been fabricated with four integrated optical fibre probes to measure light reflected from the wrist at two different wavelengths (660 and 850 nm). The ability of the sleeve to obtain a signal consistently without the need for positional adjustments is investigated.

## 2. Methodology

This section introduces the materials, the system design, the experimental setup and the experimental plan for the multichannel PPG sensor.

### 2.1. Materials

The core and cladding materials of plastic optical fibres (POF) (DB-500, Asahi Kasei, Tokyo, Japan, core diameter 480 µm and cladding thickness 20 µm) were polymethyl methacrylate (PMMA) and fluorinated polymer, respectively. The material of the buffer jacket was polyvinyl chloride (PVC). The sleeve was comprised with a yarn combination of black Nylon (600 dTex) from Fulgar (Castel Goffredo, Italy) and black Elastic (815) from Stretchline (Nottingham, UK).

### 2.2. Optical Fibre Sensor Design

**Figure 1:**
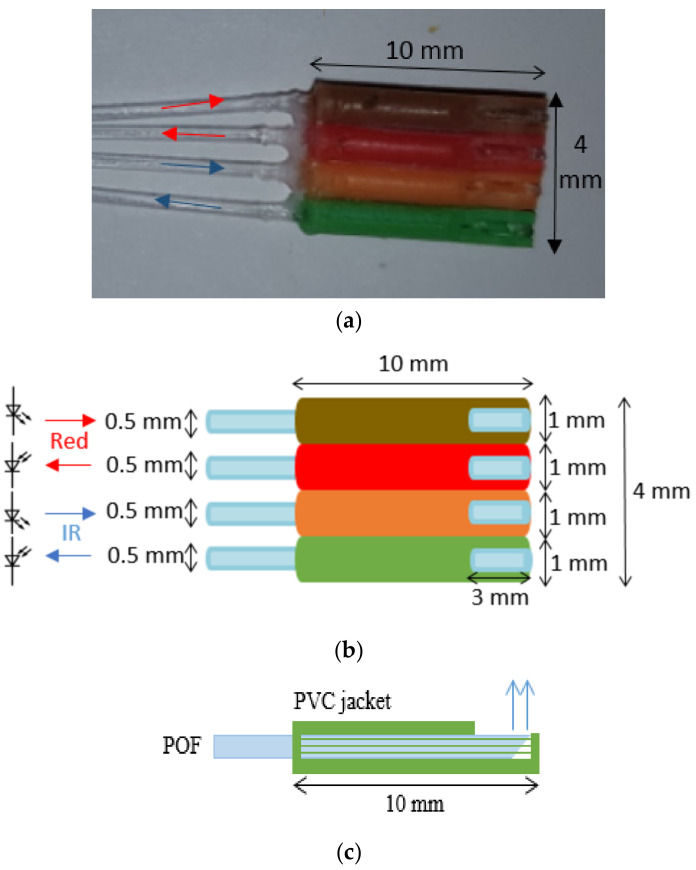
Sensor design of multichannel photoplethysmogram (PPG) sensor: (**a**) photograph of one of the four PPG sensors, (**b**) plan schematic view of one of the four PPG sensors and (**c**) side view of one sensor.

Four separate PPG sensors (named here “A, B, C and D”) were fabricated using POFs. Each of the four PPG sensors (A, B, C, D) was constructed of four optical fibres of length 100 cm. It is illustrated in Figure 1a,b. The distal end of the fibres were cleaved at a 45° angle using a sharp blade. The 45° cleave allows light to be launched into the tissue while the fibre runs parallel to the skin [26]. As can be seen from Figure 1a,b, the width of this 4 fibre sensor was 4 mm.

Two fibres were used to transmit and receive red light and two transmit and receive IR light. The tips of each of the four fibres were fixed inside four pieces (10 mm each) of a PVC jacket. The jackets were used to eliminate direct transfer of light between the receiving and illuminating fibres, which would contribute to a large DC background signal thereby interfering with R in Equation (1). Three millimetres were removed from one side of each of the jackets to expose the fibres where they would touch the skin, as shown in Figure 1b,c.

### 2.3. Sleeve Design

**Figure 2:**
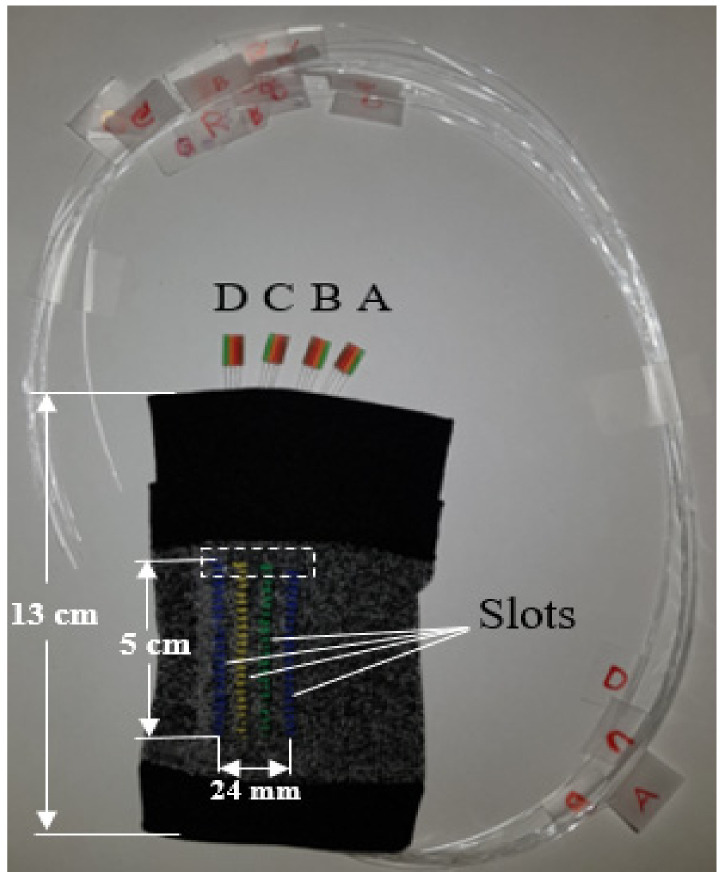
Multichannel sensors within a sleeve—for clarity the four sensors A, B, C and D are shown outside of the sleeve.

The textile sleeve was manufactured on an X-Machine (Santoni, Brescia, Italy), programmed using Santoni’s SiS_Plus Software V.0003.0001.0053. The machine was a circular seamless knitting machine with 24 individual yarn feeders, a 4-inch cylinder circumference, 144 needles, and 0.85 mm needle thickness. Each coloured textile slot on the sleeve was knitted using separate feeders with a knitting width of 4 wales across for each track. The sleeve also contained a double welt at one end allowing a fold or shelf for electronics to be held. The sleeve (Figure 2) had a circumference of 15 cm and was similar in design and comfort to elastic cuffs commonly used in knitted garments.

The four POF sensors (A, B, C and D) were fed into each slot, which holds the sensors in place without any additional fixation. In Figure 2, sensors (A, B, C and D) were extended from the textile track for better visualisation of the sleeve configuration, however, in practice, they were located within the region highlighted by the white dashed box. When the sleeve was worn, the distance from the centre of one sensor to the centre of the next sensor was ~8 mm.

### 2.4. Experimental Setup

The light sources deployed were red (wavelength λ = 660 nm, M660F1, Thorlabs, Newton, NJ, USA) and IR (λ = 850 nm, M850F2, Thorlabs, NJ, USA) LEDs with output powers of 14.5 and 6.8 mW (for multimode fibre with a 400 µm diameter) respectively. The detectors were two selective photodiodes (SPD) (EPD-660-0-0.9 and EPD-880-5-0.9, Roithner Lasertechnik, Vienna, Austria). The SPDs included an integrated filter covering the spectral range of 605–705 nm for the red channel and 800–960 nm for the IR channel with responsivities of 0.42 A/W at 660 nm and 0.55 A/W at 850 nm. The active area of each SPD was 0.62 mm^2^, and they were encased in a plastic lens. The photocurrent was converted to a voltage using a transimpedance amplifier (TIA) including an op-amp (AD8032, Analog Devices, Norwood, MA, USA) with a 2.2 nF capacitor and a 10 MΩ resistor in the feedback loop providing a low pass filter with 3 dB frequency of 7 Hz.

**Figure 3:**
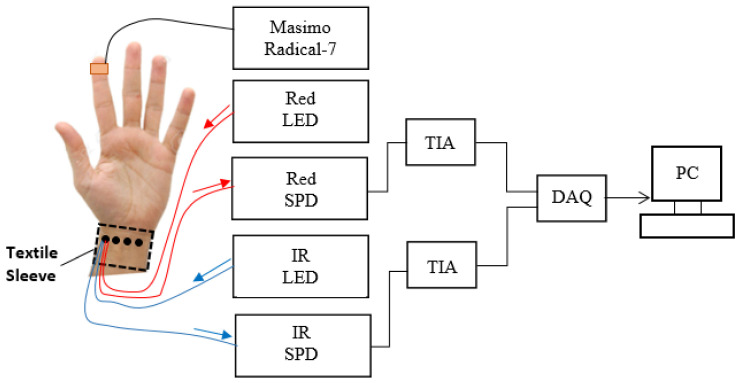
Experimental setup for multichannel PPG measurements (IR—infrared, LED—light emitting diode, SPD—selective photodiode, TIA—transimpedance amplifier, DAQ—data acquisition card, PC—personal computer).

Figure 3 shows the experimental setup for the PPG measurements. An adhesive, commercial sensor (Masimo Radical-7, Irvine, CA, USA) was attached to the index finger as an SpO_2_ reference device. In line with many clinically applied pulse oximeters, the SpO_2_ resolution was 1% with a sampling frequency of 100 Hz. For clarity, Figure 3 shows only the connections of one of four sensors. However, in practice, the textile sleeve sensor was worn on the forearm and PPG signals were detected using the four PPG sensors (A, B, C and D) located at the wrist sequentially. The fibres that were connected to the light sources and SPD were switched manually between sensors. The red and IR light travels into the skin and changes in blood volume were detected as changes in the light returned to the detectors. The outputs of the PDs were converted into a voltage via two TIAs and then converted into the digital domain by a data acquisition card (DAQ, myDAQ, 16 bits, National Instruments (NI), Austin, TX, USA) and connected to a computer via a USB connection. Two analogue channels were multiplexed into the analogue-to digital converter (ADC) channel of the DAQ. The sample frequency of the ADC was set at 2 KHz per channel and the complete system was controlled by LabVIEW software (NI, 2015 SP1). At present, the optoelectronic system uses discrete commercial components on a benchtop. However, the simplicity of the system (LEDs, photodiodes) could in future be implemented in a wearable, battery-operated, wireless optoelectronic unit.

### 2.5. Experimental Plan

#### 2.5.1. Evaluation of Transmit/Receive Properties of Each Channel

**Figure 4:**
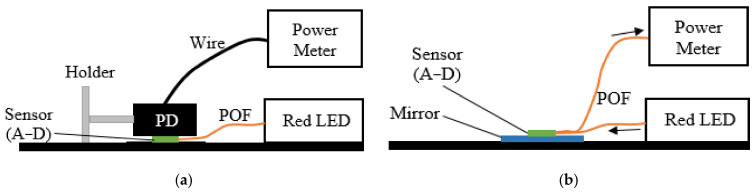
Schematic setup of measuring the light transmission (**a**) and reception (**b**).

Initially experiments were conducted to ensure the sensors provided similar light transmission and reception in each of the four channels so that systematic errors were minimal and that measured signals could be attributed to the anatomy and physiology of volunteers. To investigate the light transmission, the light output intensity of all 16 fibres (4 fibres for each sensor) was measured using an optical power meter (PM122, Thorlabs, Newton, NJ, USA) (see Figure 4a). One end of each fibre was fixed on a flat black surface, and the photo diode (PD) of the power meter was fixed on top of the fibre. The other end of the fibre was connected to the light source.

Thereafter, the light reception of all fibres was also tested by fixing each A, B, C and D sensor over a mirror (see Figure 4b). One fibre at the other end of the sensor was connected to the light source and a second fibre of the sensor was connected to the power meter. This was repeated for all 16 fibres. All readings were taken in a dark room to eliminate ambient light.

**Figure 5:**
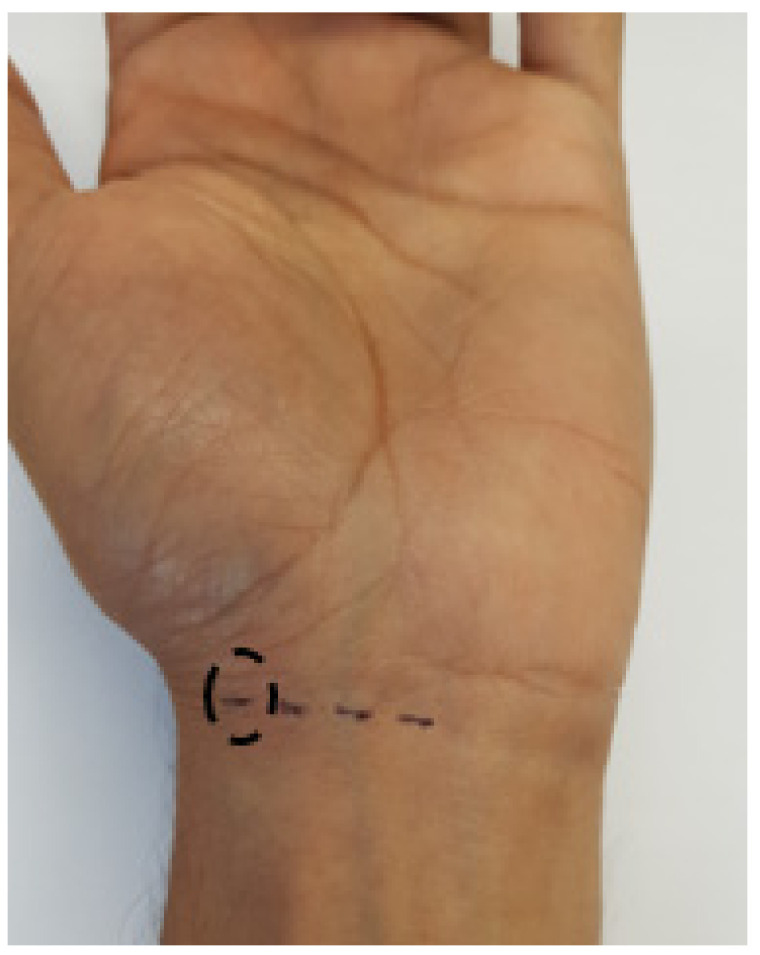
Testing all four sensors in the same position on the wrist.

Each of the four sensors (A, B, C and D, Figure 1a) was then tested in vivo in the same position on the left wrist of a single subject, as shown labelled in Figure 5 by the black circle. Although some variation was anticipated due to time delays between measurements, the purpose of this experiment was again to ensure that all sensors provided comparable transmission and reflection of light. Each recording lasted for 10 s. Low systematic errors would provide sufficient confidence to continue to a healthy volunteer study.

#### 2.5.2. In Vivo Healthy Volunteer Study

Testing on 10 healthy volunteers was approved by the Ethical Review Committee of the Faculty of Engineering at the University of Nottingham. Their wrist circumference was measured with a measuring tape. At the beginning of the experiment, the participants were asked to sit still for 5 min to relax and acclimatise to the room environment. After 5 min, the sleeve of the multichannel PPG sensor (Figure 2) was fitted on the participant’s left wrist and the commercial sensor was attached to the index finger of the participant’s left hand. PPG signals were then recorded from each of the POF sensors sequentially, to cover all four detecting points on the wrist. The light sources and SPD were switched manually between sensors. SpO_2_ signals from the commercial sensor were recorded simultaneously. Each POF sensor (A–D) was tested for 10 s (5–10 PPG beats were required and hence 10 s was adequate) for each point. Each volunteer was asked to evaluate the sleeve sensor in terms of comfort after ending the experiment. The evaluation based on a simple visual analogue scale of 0–10 [27], with anchor points 0 was “uncomfortable—I could not wear this for more than 1 min” and 10 was “extremely comfortable—I could wear this all day”.

### 2.6. Signal Quality

In order to quantify the PPG signal quality, two characteristics were measured, namely, Perfusion Index (PI) and signal to noise ratio (SNR). PI is a parameter that refers to the ratio of the pulsatile signal (AC component) to the nonpulsatile signal (DC component), expressed as $\frac{\mathrm{AC}}{\mathrm{DC}}\times 100\%$ [28]. PI is a normalised estimation of the pulse’s strength and ranges from 0.02% (weak pulse) to 20% (strong pulse) [29].

**Figure 6:**
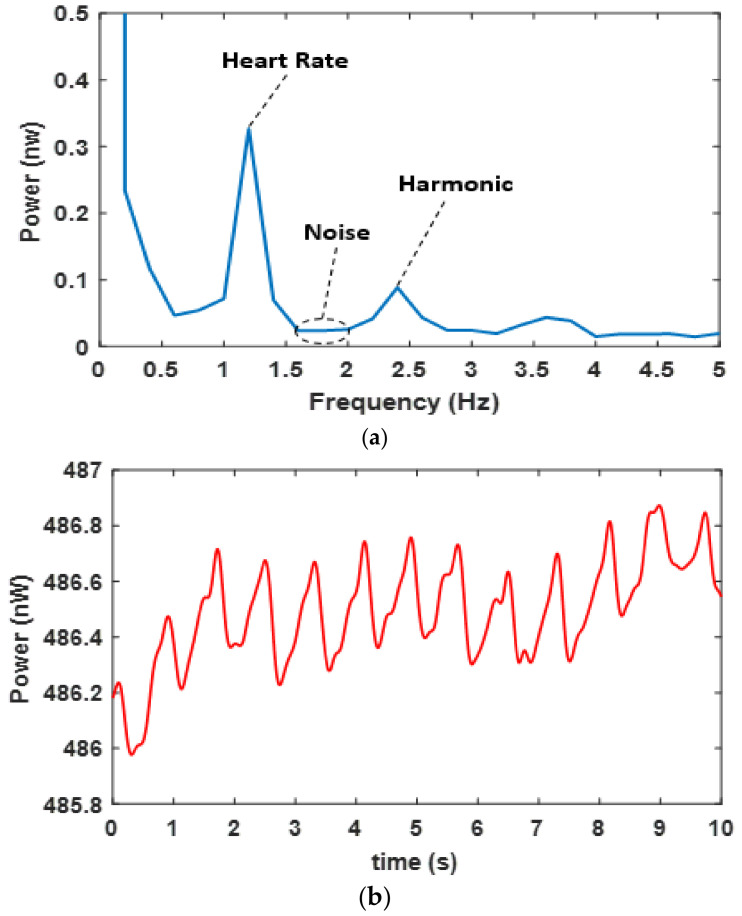
(**a**) Average (over 5 s data) FFT of red PPG signal showing the signal region (heart rate) and noise region for calculating SNR (**b**) signal in the time domain.

The signal to noise ratio (SNR) of each PPG signal was calculated in the frequency domain using the Fast Fourier Transform (FFT). The heart rate of a normal adult is approximately 1.2 Hz, and its harmonic is 2.4 Hz. The noise was measured from the silent region (that does not include any physiological parameters such as breathing or heartbeat activities), in this case, the average value of the region between 1.7 and 2 Hz was selected [30]. As an example, Figure 6a shows these features in the FFT of the red PPG (Figure 6b) taken over 5 s of data, the FFT sampling rate was 2 KHz and the frequency resolution (bin width) was 0.2 Hz.

## 3. Results

### 3.1. Evaluation of Transmit/Receive Properties of Each Channel

As discussed in Section 2.5.1, as each channel is manufactured by hand, it is important to characterise the variability between each channel in terms of the transmission (Figure 4a) of the light to the tissue surface and reception (Figure 4b) of the light from the tissue. Low system variability in each channel would provide confidence that any variability during in vivo measurements is due to the relative positioning of the sensors and blood vessels and not due to systematic errors.

**Figure 7:**
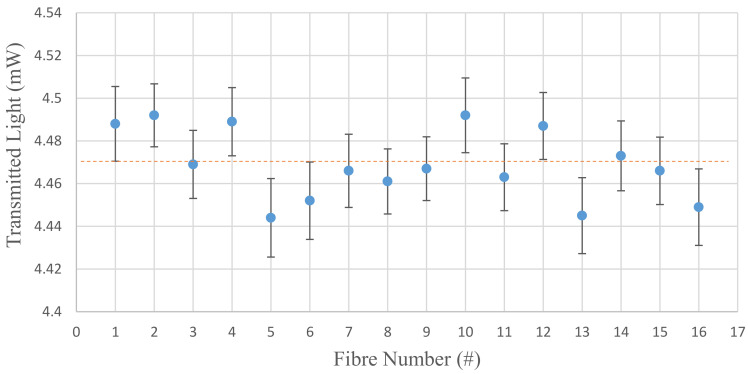
Light transmission power of all 16 fibres used in the multichannel sensor using the configuration in Figure 4a.

Figure 7 shows the transmitted light output power from each of the 16 fibres measured in the setup of Figure 4a. The error bars are the standard deviation of N (10) measurements about the mean for that fibre. The mean output values of light transmission from these 16 fibres were all between 4.44 and 4.5 mW with a mean (orange dashed line) and standard deviation of 4.47 ± 0.02 mW.

**Figure 8:**
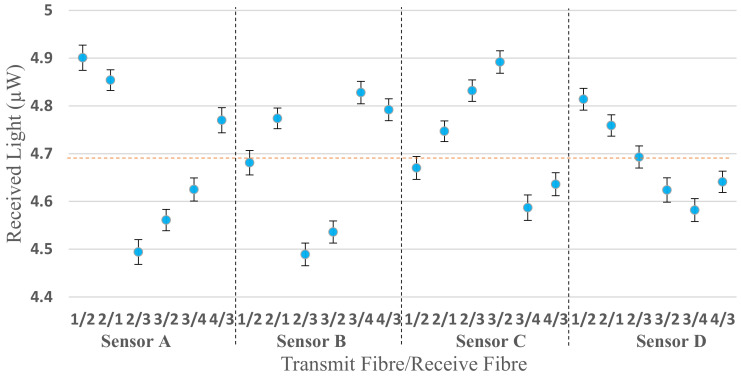
Received light of each fibre deployed in the multichannel sensor using the mirror configuration in Figure 4b. The pairs, e.g., 1/2 indicates the transmit fibre first and receive fibre second for each sensor A–D.

Similar characterisation was performed for different transmit/receive pairs using the configuration shown in Figure 4b. Figure 8 shows the reflected light from the mirror of each fibre of all four sensors, e.g., sensor A (1/2) in the figure means that the sensor is A, and the light was sent through fibre number 1 and collected from fibre number 2. The average value of the received light is 4.70 ± 0.12 µW.

The IBM SPSS Statistics 24 software was used to evaluate statistically significant differences between all sensors (A, B, C and D). The normality test using Kolmogorov–Smirnov shows that all four sensors’ data are non-normally distributed (Table 1). As a result, the Kruskal–Wallis test was applied to the original A, B, C and D data in Figure 8 and no significant difference was observed (*p* = 0.223).

Table 2 shows preliminary results of testing all four sensors (A, B, C and D) when placed in the same position on the wrist of a single subject (see Figure 5). This preliminary experiment was conducted to investigate where different responses in the channels affected PPG measurements before progressing to a healthy volunteer study. Despite some variability due to manual positioning of the sensors and a short (a few seconds) time lag between readings, measurable signals could be obtained for both red and IR PPGs. These three sets of test measurements provided sufficient confidence to proceed to a healthy volunteer study.

### 3.2. In Vivo Healthy Volunteer Study

**Figure 9:**
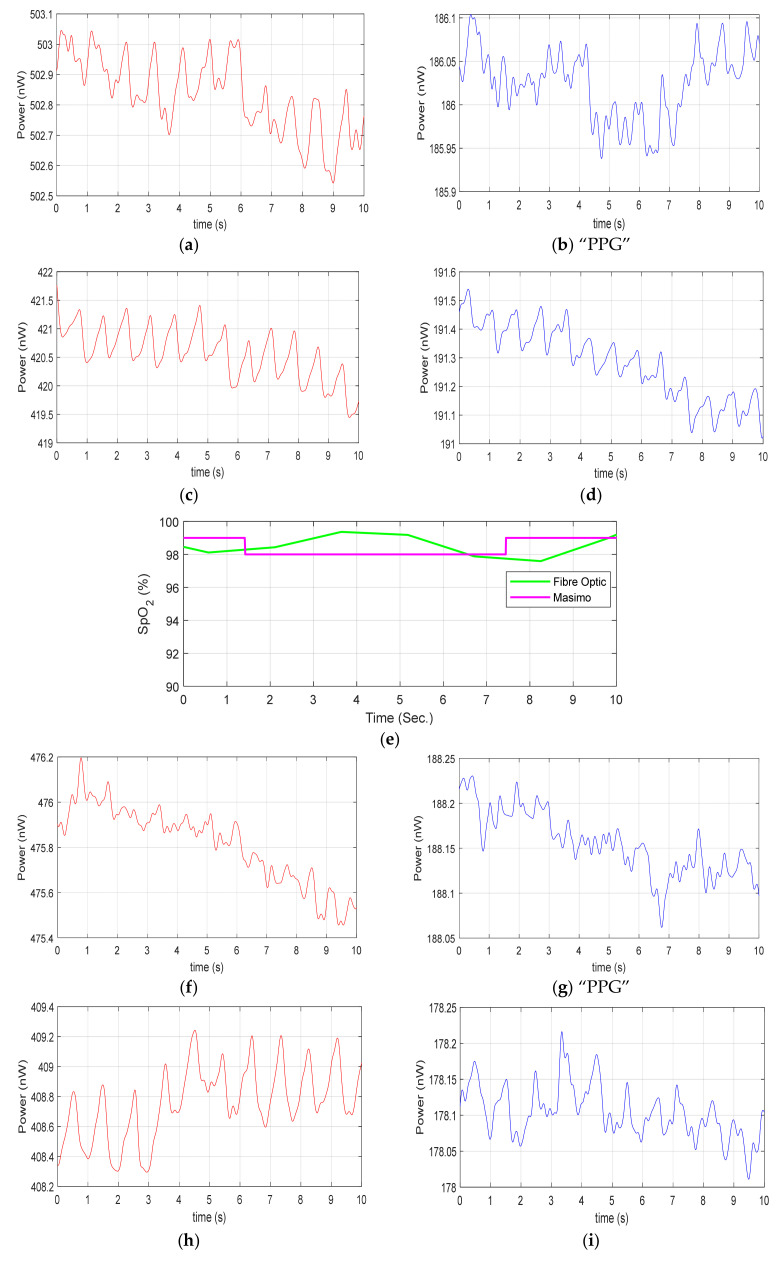

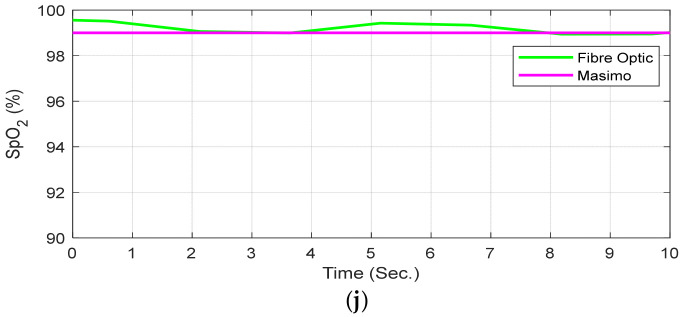
Healthy volunteer (Participant P9) example traces, (**a**) red channel PPG, sensor A; (**b**) IR channel “PPG,” sensor A; (**c**) red channel PPG, sensor B; (**d**) IR channel PPG, sensor B; (**e**) SpO_2_ fibre sensor B and Masimo reference device; (**f**) red channel PPG, sensor C; (**g**) IR channel “PPG,” sensor C; (**h**) red channel PPG, sensor D; (**i**) IR channel, sensor D; (**j**) SpO_2_ sensor D and Masimo reference device. Note: Those traces with “PPG” designation indicate that a PPG cannot be clearly seen and hence an SpO_2_ calculated trace is not presented.

The multichannel (A, B, C, D) sensor was then tested on 10 healthy volunteers’ wrists. The DC red light power illuminating the proximal end of each POF was 14.5 mW, the DC light power illuminating the skin at the fibre tip of 45° angle was 4.47 ± 0.02 mW (Figure 9). The red and IR signals were recorded for each sensor (A, B, C, D), and the SpO_2_ level was measured when both red and IR PPG were clearly visible. Figure 9 shows the traces for participant P9. For example, Figure 9b,g did not clearly show a visible PPG and hence no SpO_2_ was calculated. This figure presents the results from one participant; the other volunteers’ results are presented in Appendix A. Figure 9e shows SpO_2_ calculated from the red and IR PPGs of sensor B and Masimo (magenta trace) were monitored simultaneously. Figure 9j shows SpO_2_ calculated from the red and IR PPGs of sensor D and Masimo (magenta trace), monitored simultaneously. The resolution of the Masimo device is 1%, SpO_2_ and so these data appear quantized.

Table 3 tabulates the same results from all four sensors on the same healthy volunteer as shown in Figure 9a–j, i.e., participant P9; the other volunteers’ results are presented in Appendix B. For this participant, as we have seen that two sensors (D and B) provide red and IR PPG visible traces allowing SpO_2_ calculations to be compared to a commercial device. However, sensor A was only able to detect red PPG, while sensor C was unable to obtain any PPG signals in either red or IR channel. It is worth noting that DC light levels are comparable for each sensor and each channel has low systematic variability (Section 3.1) and so lack of a PPG signal is a positional effect, not simply a light collection issue. To enable comparison, the SpO_2_ average value with the commercial pulse oximeter and POF sensor is calculated over 10 s of data. The SpO_2_ resolution of the commercial oximeter available to us is 1% and a more accurate comparison could be achieved with a higher resolution pulse oximeter in future.

Table 4 shows the readings of all sensors that obtained SpO_2_ levels across all 10 participants. At least one POF sensor was able to detect SpO_2_ levels without any sensor positional adjustment for each participant. For two volunteers (participant number (P#): 4 and 9), the average SpO_2_ level was measured by two different sensors. The detecting sensor varied between participants due to variations in sensing point, artery structure and wrist circumference (16.1 ± 0.6 cm) between volunteers.

**Figure 10:**
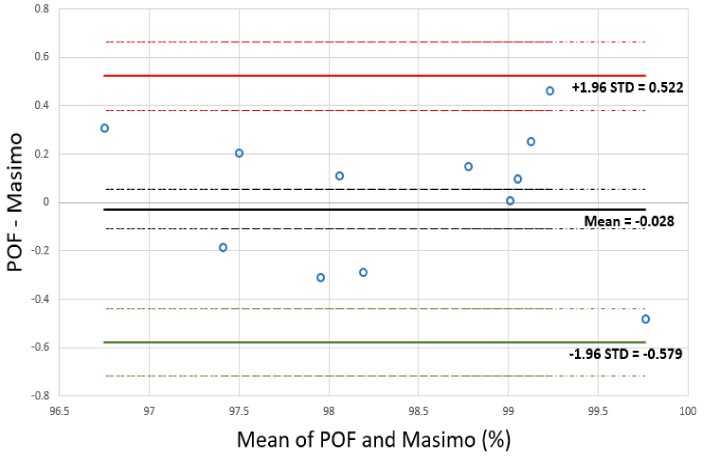
Bland–Altman plot of the difference of Masimo and multichannel SpO_2_ PPG sensor against the mean of Masimo and multichannel SpO_2_ PPG. Mean difference and limits of agreements (LOAs) are shown as solid lines while dotted lines show the confidence limits of each.

The calculated SpO_2_ readings from the multichannel PPG sensors and the reference (Masimo) sensor for the 12 sensor positions listed in Table 4 are compared in Figure 10. This figure presents a Bland–Altman plot that plots the difference of Masimo SpO_2_ and POF SpO_2_ against the mean of Masimo SpO_2_ and POF SpO_2_ devices [31,32]. The plot contains 12 SpO_2_ measurements from 10 volunteers. The mean (µ) of the differences is −0.028% (solid black line), the standard deviation (STD) of the differences is 0.28 and the limits of agreements (LOAs) are:

$$\begin{array}{c} -\mathrm{LOA}\;=\;µ\;-\;1.96\mathrm{STD}\;=\;-0.028\;-\;\left(1.96\;\times \;0.28\right)\;=\;-0.58\;\left(\mathrm{solid}\;\mathrm{green}\;\mathrm{line}\right) \end{array}$$

$$+\mathrm{LOA}\;=\;µ\;+\;1.96\mathrm{STD}\;=\;-0.028\;+\;\left(1.96\;\times \;0.28\right)\;=\;0.52\;\left(\mathrm{solid}\;\mathrm{red}\;\mathrm{line}\right).$$

These LOAs, shown clearly in Figure 10 (Red and green solid lines), are the range over which 95% of differences between the two sensors lie. The standard error of the µ and the 95% LOAs were calculated to determine if the sample size was large enough for a meaningful comparison of the results [31]. The standard error of the mean is $\sqrt{STD^{2}/n}$ = 0.081 (dashed black lines) and the standard error of the LOA is $\sqrt{3STD^{2}/n}$ = 0.14 (dashed red and green lines), where n is the number of samples.

The results of the comfort evaluation of the sleeve sensor on the scale from 0 to 10 had a mean of 8.8 and median of 9. Although a very simple evaluation, this indicates that the sleeve sensor was comfortable for users during the experiment.

## 4. Discussion

This paper has investigated whether a multichannel approach of 16 fibres over 24 mm can provide a reliable PPG measurement. It was demonstrated that at least one fibre pair could obtain a PPG, therefore, allowing uninterrupted measurement of SpO_2_ to be implemented in such a wearable device. For example, for participant P9 shown in Figure 9, Sensor A only obtained a red PPG signal. Sensors B and D were able to detect both red and IR PPG and could thus be used to measure SpO_2_. Sensor C did not provide any PPG signals. This 100% reliable PPG detection was possible in all 10 subjects. Further, these results demonstrate that sensor position is important for obtaining high quality PPG signals, and this is likely to be related to whether the sensor lies near a major vessel. The depth of the main vessels and their branches (superficial vessels) that are running under the wrist skin of ranges between 1 and 3 mm [33], while the penetration depth of red and IR wavelengths in skin tissue is typically up to ~5 mm depending on the source detector separation [34].

Table 5 is a comparative table of the relevant studies on multichannel PPG in comparison with the sleeve sensor in terms of sensor configuration, sensing points, number of PD and LED and LED power.

As the optical power of IR (6.8 mW) was lower than red (14.5 mW), there was lower contrast in the IR signals and many of the sensors detected only a red PPG signal, as shown in Appendix A. This also reflects the fact that the body absorbs light at different wavelengths to varying degrees and to different depths in different positions [35]. Table A1 in Appendix B presents the results from all four sensors for all 10 participants, showing which sensors detect a PPG at both wavelengths and those that detect a PPG at only one wavelength and finally those which failed to detect a PPG at all. The overall result was that a PPG for both red and IR was always detected in all 10 subjects.

We envisage that this multichannel wrist sensor could easily be incorporated into a typical shirt where the user could wear the item normally without repositioning the wristband. As garments are produced, further sophisticated comfort evaluation tools can be used such as blind subjective evaluation and visual subjective evaluation techniques [36]. Alternatively, this multichannel technique can be applied favourably in many other settings such as within a cap for the monitoring of newborns [37] adults or on a pregnant mother’s abdomen, e.g., In such settings, the reliable measurements of a newborn or foetus without the necessity of repositioning the device opens up considerable opportunities for the healthcare community [16,37].

Conducting the investigation required light sources to be manually switched between sensors. However, in future, fully multiplexed designs would be incorporated with multiple sources and detectors in a compact autonomous electronic unit. The optoelectronics is currently large and sits on a benchtop but in future, this can be made into a compact, battery-operated wireless unit. The Thorlabs LEDs could be replaced by a small LEDs (e.g., 606-CMD333URC2, 782-VSLY5850, Mouser Electronics, High Wycombe, UK) and implemented on the same PCB as photodiodes and TIAs. An ADC chip (e.g., ADS1115, Texas Instruments, Dallas, TX, USA) could be used instead of the DAQ. The data could be transmitted to the PC via wireless using a Bluetooth module. The whole system can be housed in a small box (estimated size ≈ 45 mm × 35 mm) and incorporated in the welt of the sleeve of a shirt for ambulatory monitoring. This will allow field testing of the device, e.g., during exercise. This would subject the sensors to other effects such as skin moisture changing skin reflectivity. This is a problem for all PPG sensors mitigated by taking the “ratio of ratios” R (Equation (1)) but may be further reduced by the multichannel configuration. In addition, light coupling at the tissue interface was inefficient and hence methods to increase the collection of reflected light by say adding micro-optic lenses to the received fibres is required to further improve the system performance.

## 5. Conclusions

Photoplethysmography for heart rate and oxygen saturation monitoring can be performed in an unobtrusive and comfortable form using optical fibres integrated into textiles. However, reflectance PPG measurement reliability depends on the position on the skin surface and is highly dependent upon sensor/skin movement. We have demonstrated that a multichannel approach can be used to obtain a highly reliable measurement that can be integrated into a textile such as a shirt specifically around the wrist.

In tests of 10 healthy volunteers, the multichannel sensor in the textile sleeve allowed a PPG signal to be obtained 100% of the time without adjustment of its position offering the potential to be deployed in many locations of the human body. An SpO_2_ signal was therefore obtained from at least one point on the wrist for every volunteer. Comparison of oxygen saturation results between the multichannel sensor and a reference commercial sensor showed that the mean difference between the two sensors’ readings was −0.03 and the upper LOA was 0.52 and the lower LOA was −0.58.

## Figures and Tables

**Table 1:** Kolmogorov–Smirnov (normal distribution) and Kruskal–Wallis tests between all four sensors (A, B, C and D).

Sensor	*p*-Value	
	Kolmogorov–Smirnov Test (>0.05–Normal Distribution) (≤0.05–Non-Normal Distribution)	Kruskal–Wallis Test (>0.05–No Significant Difference) (≤0.05–Significant Difference)
A	0.01	0.223
B	<0.001	
C	0.047	
D	0.024	

**Table 2:** Readings from all four sensors (A–D) tested in the same position on the left wrist; photoplethysmogram (PPG) peak to peak average (P-P_ave_), Perfusion Index (PI) average and signal to noise ratio (SNR) for both wavelengths, and the average calculated oxygen saturation (SpO_2_) was obtained over a 1.5 s window.

Sensor	Signal	P-P_ave_ (nW)	PI (%)	SNR	SpO_2_ (%)
A	Red	0.3	0.08	5.27	98.2
	IR	0.15	0.07	6.28	
B	Red	0.32	0.07	6.50	99
	IR	0.16	0.08	6.52	
C	Red	0.31	0.06	5.95	98.5
	IR	0.15	0.07	6.34	
D	Red	0.3	0.08	6.18	98.3
	IR	0.17	0.09	6.77	

**Table 3:** Summary of readings from all four sensors (A, B, C and D) from four different positions on the left wrist of healthy volunteer P9; non-pulsatile component (DC) level (nW), the average PPG peak to peak (P-P_ave_), Perfusion Index (PI) average and SNR for both wavelengths; and the average SpO_2_ levels from both the plastic optical fibre (POF) and Masimo sensors.

Sensor	Signal	DC Level (nW)	P-P_ave_ (nW)	PI (%)	SNR	Average SpO_2_ (%)	
						POF	Masimo
A	Red	502.8	0.20	0.04	6.63	–	98.9
	IR	186.0	–	–	–		
B	Red	420.7	0.84	0.19	7.19	98.1	98.3
	IR	191.3	0.15	0.08	4.17		
C	Red	475.8	–	–	–	–	99
	IR	188.1	–	–	–		
D	Red	408.9	0.52	0.13	5.98	99.5	99
	IR	178.1	0.10	0.06	4.29		

**Table 4:** Summary of results from all sensors that provided an SpO_2_ value from all 10 volunteers at different positions on the wrist; non-pulsatile component (DC) level (nW), the average PPG peak to peak (P-P_ave_), Perfusion Index (PI) average and SNR for both wavelengths; and the average SpO_2_ levels reported by both POF and Masimo sensor.

P#	Sensor	Signal	DC Level (nW)	P-Pave (nW)	PI (%)	SNR	Average SpO_2_ (%)	
							POF	Masimo
1	D	Red	405.0	0.97	0.24	11.59	98.1	98
		IR	193.6	0.13	0.06	6.32		
2	B	Red	393.5	1.1	0.28	3.87	96.9	96.6
		IR	201.5	0.9	0.04	2.35		
3	D	Red	311.1	0.53	0.17	5.89	97.6	97.4
		IR	188.1	0.11	0.06	6.39		
4	D	Red	385.1	0.26	0.07	6.75	99.1	99
		IR	185.4	0.12	0.07	6.94		
	B	Red	455.6	0.28	0.06	5.28	99	99
		IR	203.3	0.09	0.05	7.07		
5	B	Red	477.7	0.43	0.09	10.96	97.8	98.1
		IR	193.6	0.087	0.04	7.51		
6	D	Red	428.5	2.73	0.64	4.1	99.3	99
		IR	185.8	0.13	0.07	4.47		
7	A	Red	437.0	0.32	0.07	7.33	99.5	100
		IR	191.6	0.11	0.06	5.99		
8	C	Red	385.4	0.52	0.13	5.80	98.9	98.7
		IR	180.3	0.11	0.06	5.30		
9	D	Red	408.8	0.52	0.13	5.98	99.5	99
		IR	178.1	0.10	0.06	4.29		
	B	Red	420.7	0.84	0.19	7.19	98.1	98.3
		IR	191.3	0.15	0.08	4.17		
10	D	Red	385.3	0.24	0.06	6.86	97.3	97.5
		IR	182.3	0.1	0.05	6.27		

**Table 5:** Summary of the relevant studies on multichannel PPG.

Configuration	Sensing Point	PD/LED	LED Power (mW)	References
Textile sleeve	Wrist	2/2 (1 red and 1 IR)	14.5 red, 6.8 IR	This study
Ear-worn platform	Ear	3/2 IR	2	Wang et al. [18]
Wrist watch	Wrist	4/4 IR	2	Lee et al. [19]
Elastic headband	Forehead	1/6 (3 red and 3 IR)	–	Mendelson et al. [20]
Electronic patch sensor	Forehead, wrist, head	1/16 (4 red, 4 IR, 4 green, 4 yellow)	–	Alzahrani et al. [21]

**Figure A1:**
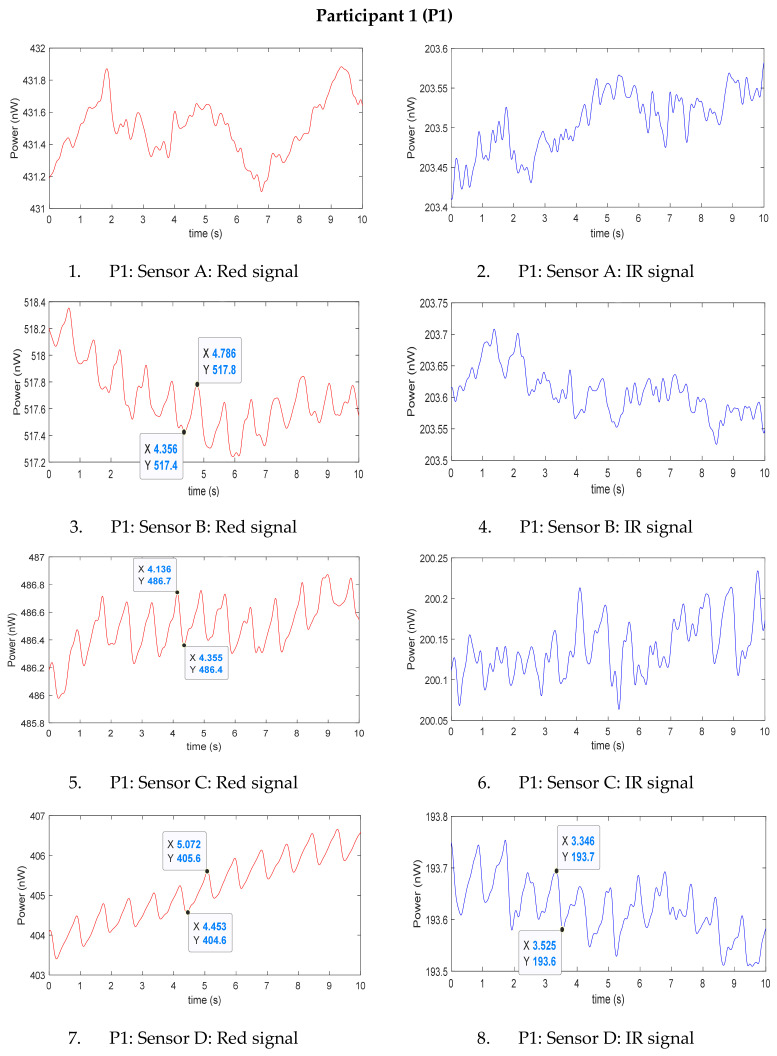

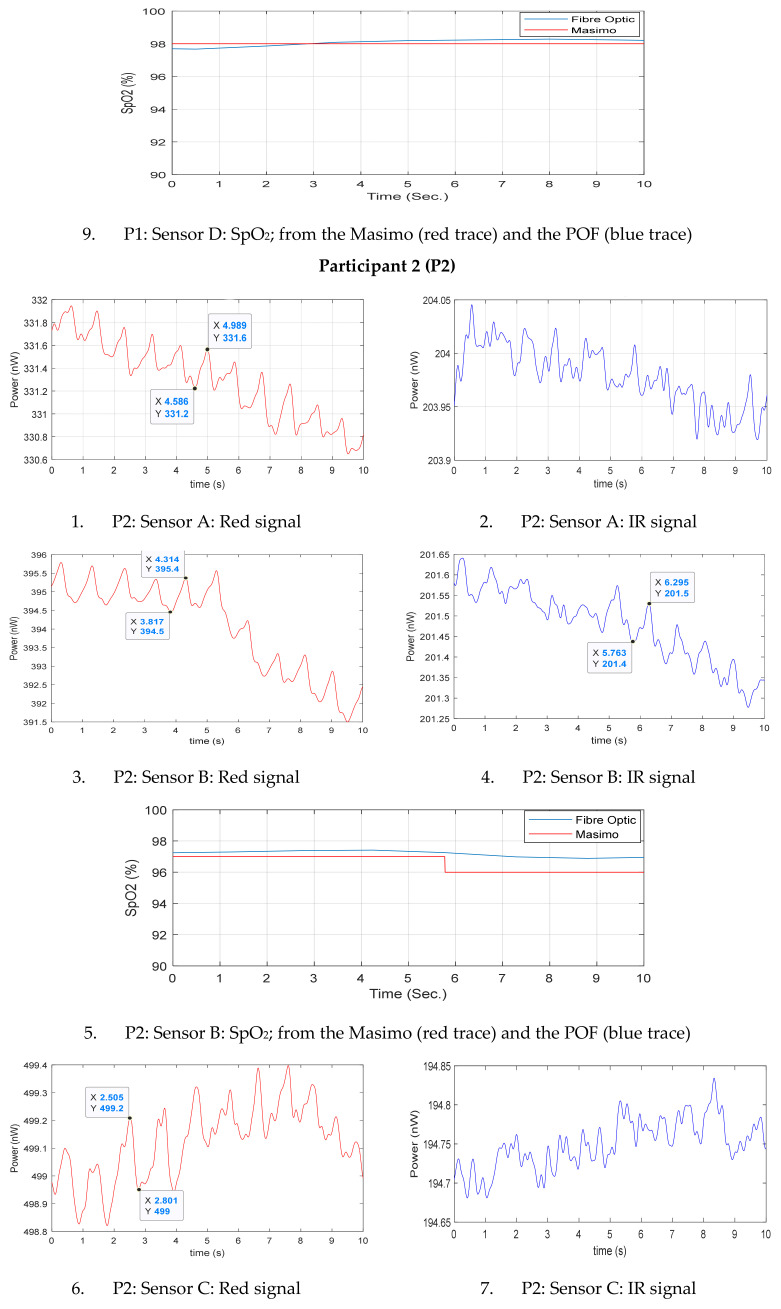

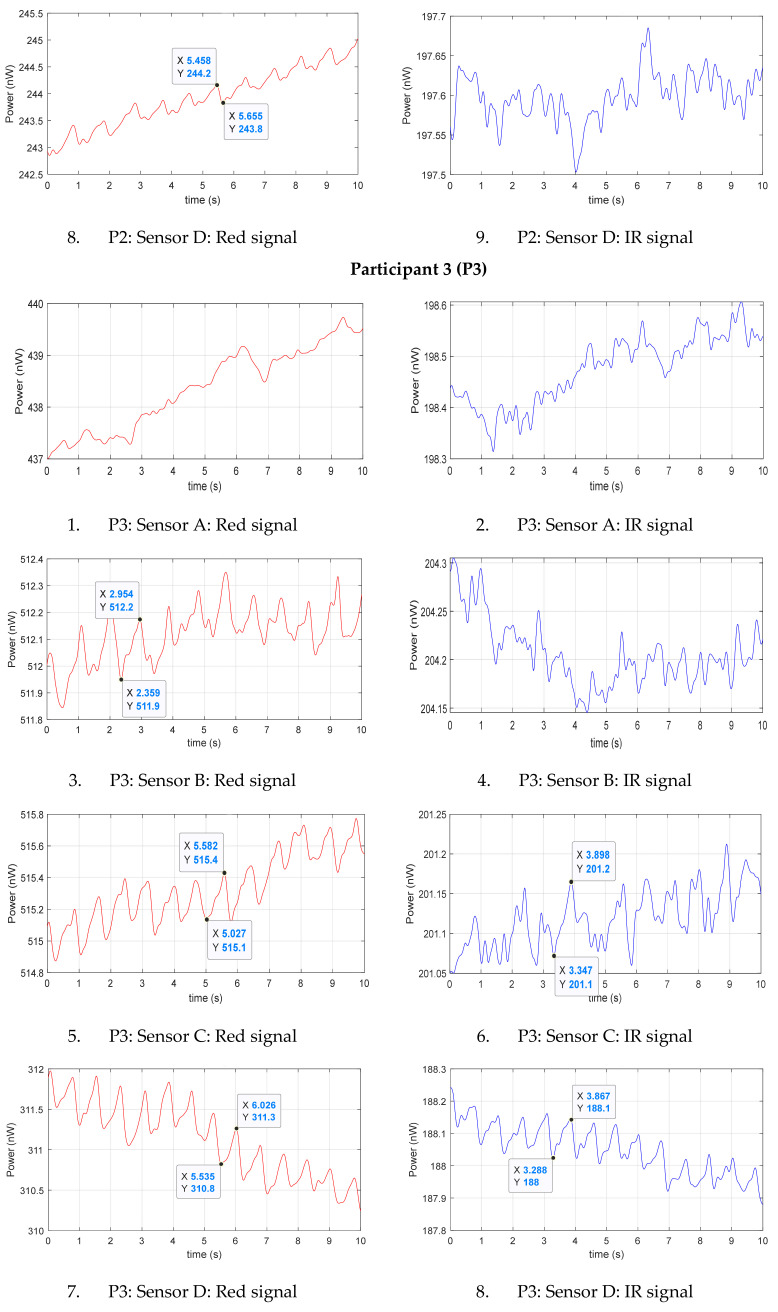

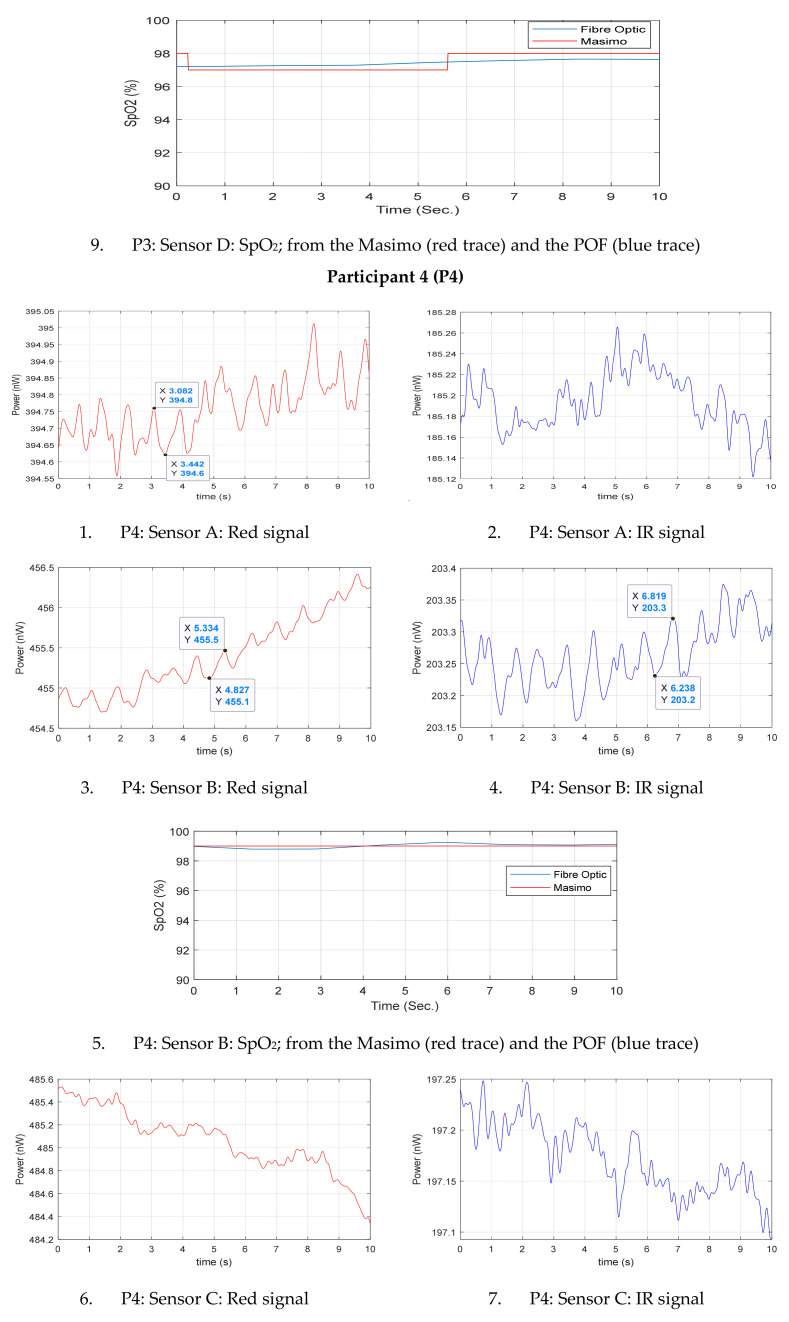

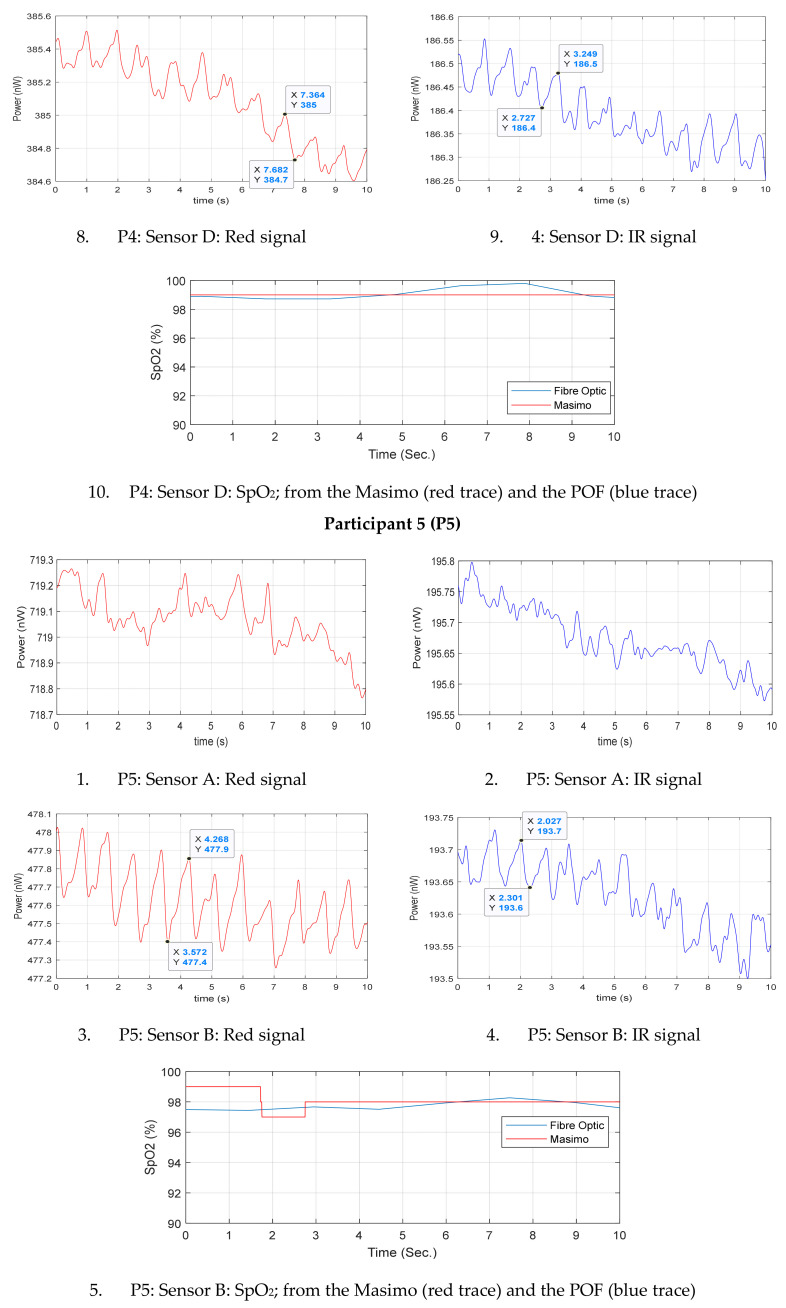

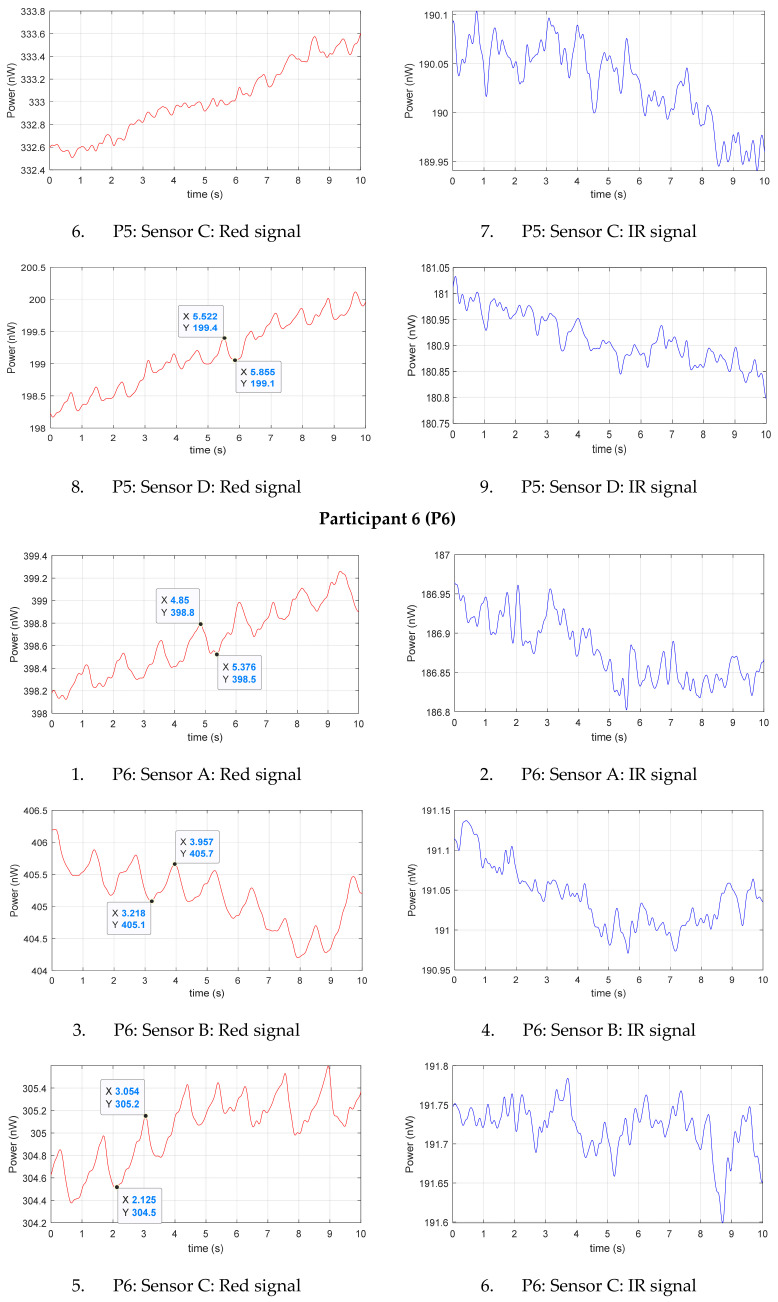

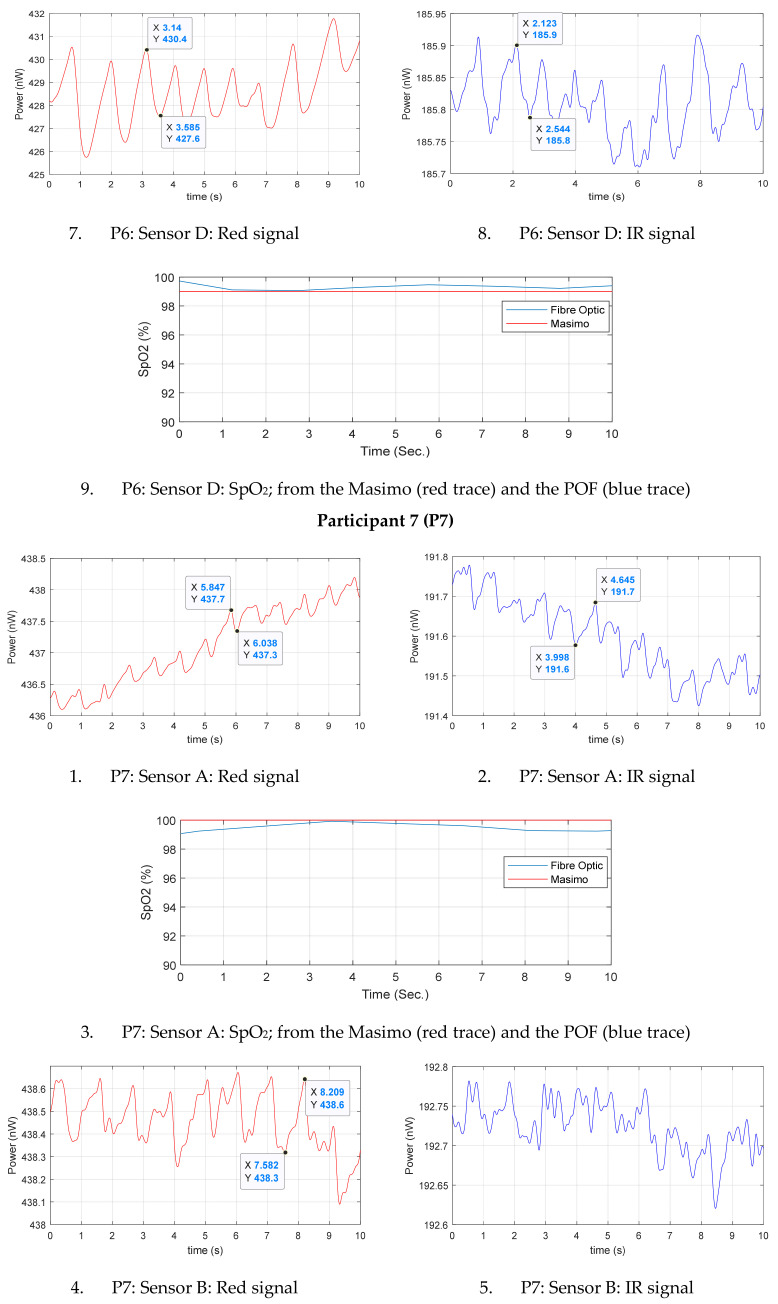

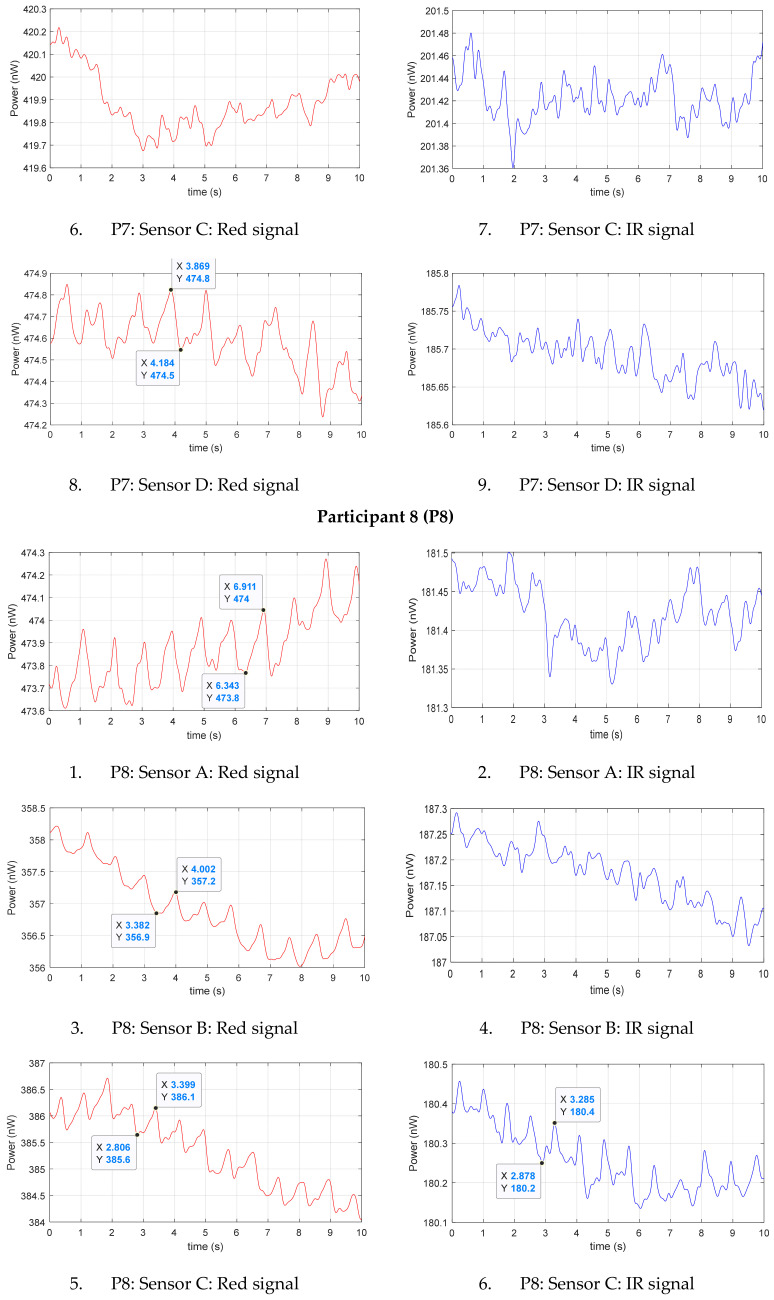

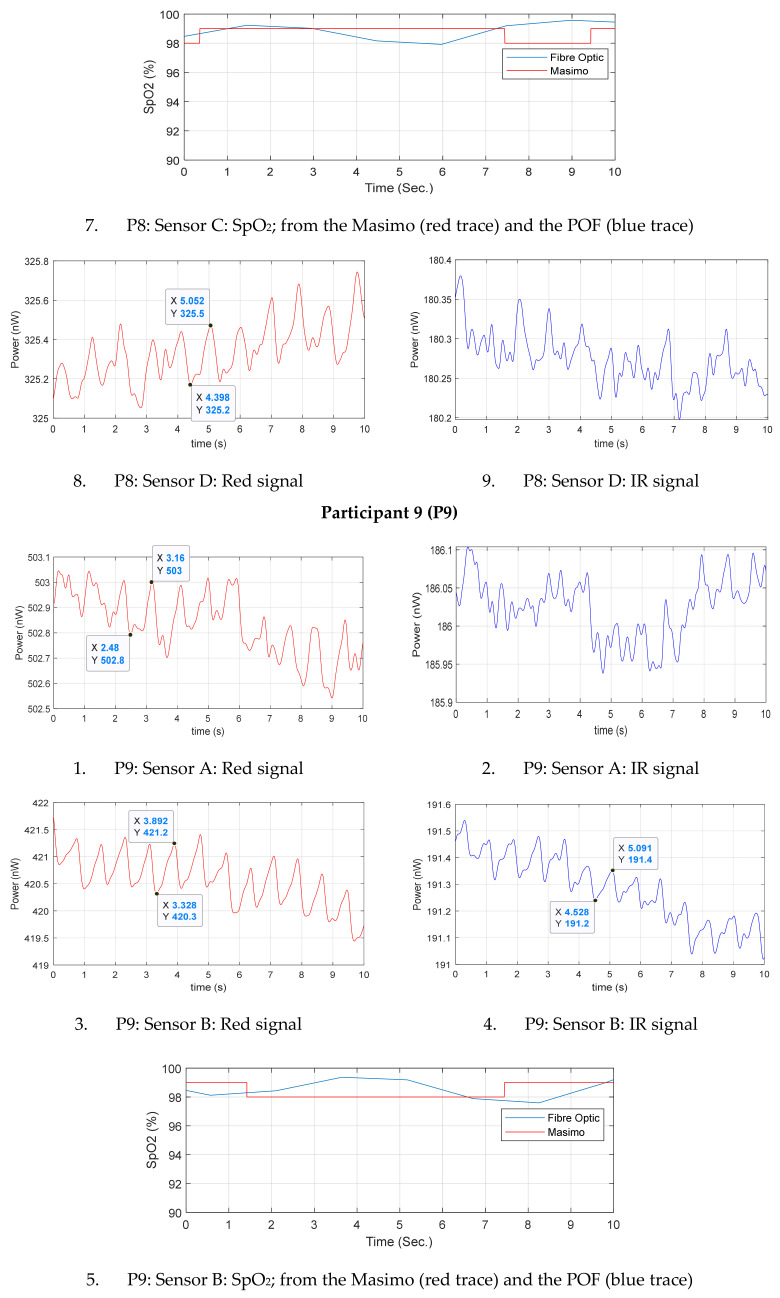

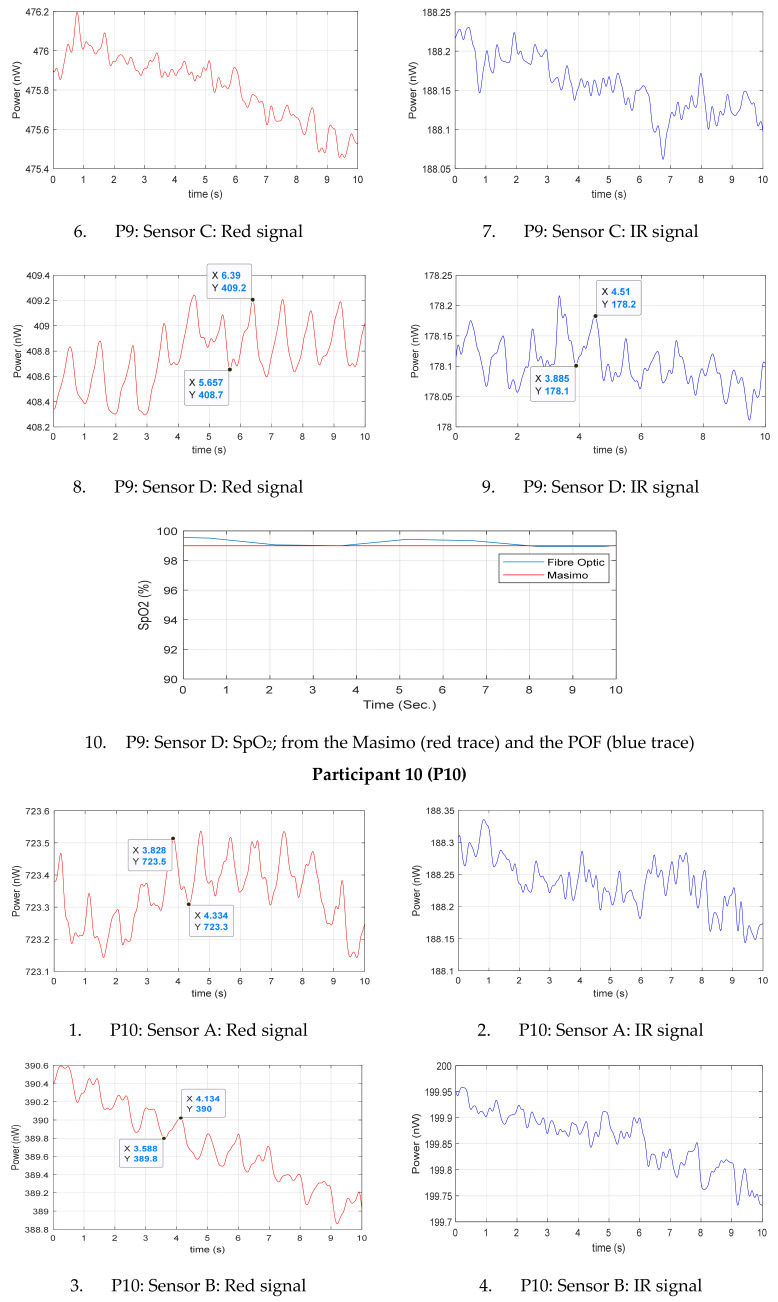

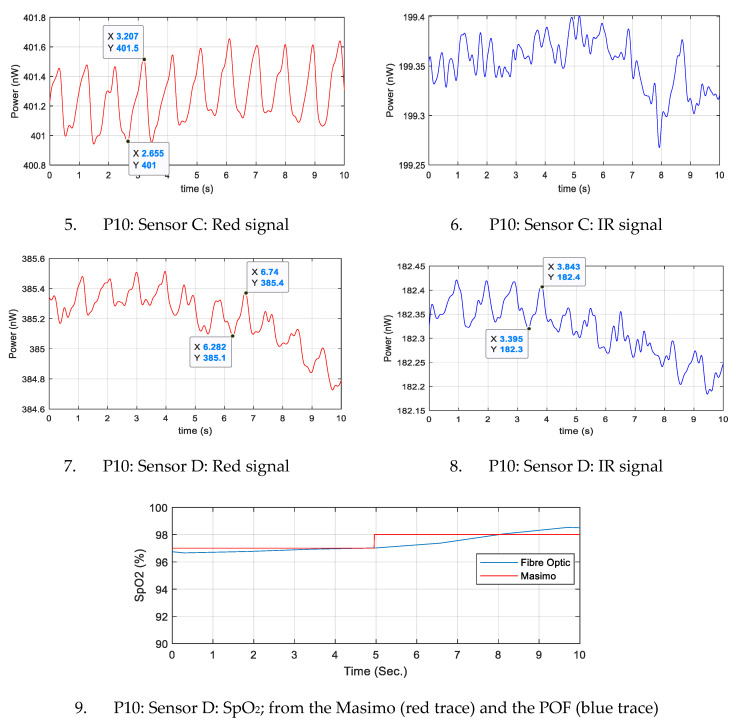
Red and IR signals from each sensor (A, B, C and D) of all participants (1–10), and oxygen saturation (SpO_2_) readings from the sensors that provided both photoplethysmogram (PPG) signals.

## Appendix A

Appendix A and Appendix B show the results of the multichannel PPG experiments conducted on the healthy volunteers on their wrist. Appendix A illustrates the red and IR signals from each sensor for all participant (P#). SpO_2_ level is shown if PPGs are detectable at both wavelengths. Appendix B summarizes parameters from all results of A1; DC level, the average PPG peak to peak (P-P_ave_), Perfusion Index (PI) and SNR from both wavelengths and the average SpO_2_ level of POF and the commercial reference device.

## Appendix B

**Table A1:** Summary of readings from all four sensors (A–D) from four different positions on the left wrist of all participants (P#); DC level, peak to peak average (P-Pave), Perfusion Index (PI) average and signal to noise ratio (SNR) for each type of light; and the average oxygen saturation (SpO_2_) levels from both the plastic optical fibre (POF) and Masimo sensors.

P#	Sensor	Signal	DC Level (nW)	P-Pave (nW)	PI (%)	SNR	Average SpO_2_ (%)	
							POF	Masimo
1	A	Red	431.4	–	–	–	–	98
		IR	203.5	–	–	–		
	B	Red	517.7	0.4	0.08	7.99	–	99
		IR	203.6	–	–	–		
	C	Red	486.4	0.33	0.07	12.79	–	99.1
		IR	200.15	–	–	–		
	D	Red	405	0.97	0.24	11.59	98.1	98
		IR	193.6	0.13	0.06	6.32		
2	A	Red	331.2	0.36	0.11	6.31	–	97.1
		IR	203.97	–	–	–		
	B	Red	393.5	1.1	0.28	3.87	96.9	96.6
		IR	201.45	0.9	0.04	2.35		
	C	Red	499.15	0.22	0.045	4.99	–	97.3
		IR	194.75	–	–	–		
	D	Red	244	0.475	0.19	3.94	–	97
		IR	197.6	–	–	–		
3	A	Red	438.5	–	–	–	–	97.7
		IR	198.46	–	–	–		
	B	Red	512.1	0.21	0.04	3.95	–	98.3
		IR	204.2	–	–	–		
	C	Red	515.3	0.32	0.06	9.54	–	98
		IR	201.13	–	–	–		
	D	Red	311.1	0.53	0.17	5.89	97.6	97.4
		IR	188.05	0.11	0.06	6.39		
4	A	Red	394.70	0.22	0.055	4.44	–	98
		IR	185.18	–	–	–		
	B	Red	455.6	0.28	0.06	5.28	99	99
		IR	203.26	0.09	0.05	7.07		
	C	Red	485.90	–	–	–	–	98
		IR	197.16	–	–	–		
	D	Red	385.10	0.26	0.07	6.75	99.1	99
		IR	185.40	0.12	0.07	6.94		
5	A	Red	719	–	–	–	–	97.7
		IR	195.65	–	–	–		
	B	Red	477.65	0.43	0.09	10.96	97.8	98.1
		IR	193.62	0.087	0.04	7.51		
	C	Red	333.10	–	–	–	–	98.2
		IR	190.03	–	–	–		
	D	Red	199.30	0.34	0.17	2.41	–	99
		IR	180.90	–	–	–		
6	A	Red	398.7	0.24	0.06	5.50	–	98.9
		IR	186.87	–	–	–		
	B	Red	405.2	0.66	0.16	5.36	–	99
		IR	191.04	–	–	–		
	C	Red	305	0.52	0.17	5.15	–	100
		IR	191.7	–	–	–		
	D	Red	428.50	2.73	0.64	4.1	99.3	99
		IR	185.80	0.13	0.07	4.47		
7	A	Red	437	0.32	0.07	7.33	99.5	100
		IR	191.6	0.11	0.06	5.99		
	B	Red	438.48	0.28	0.06	3.95	–	99
		IR	192.72	–	–	–		
	C	Red	419.9	–	–	–	–	100
		IR	201.42	–	–	–		
	D	Red	474.60	0.29	0.06	3.20	–	100
		IR	185.70	–	–	–		
8	A	Red	473.88	0.29	0.06	8.04	–	98
		IR	181.40	–	–	–		
	B	Red	356.90	0.44	0.12	5.49	–	98
		IR	187.15	–	–	–		
	C	Red	385.40	0.52	0.13	5.80	98.9	98.7
		IR	180.26	0.11	0.06	5.30		
	D	Red	325.40	0.37	0.11	4.29	–	97.4
		IR	180.25	–	–	–		
9	A	Red	502.84	0.20	0.04	6.63	–	98.9
		IR	186	–	–	–		
	B	Red	420.7	0.84	0.19	7.19	98.1	98.3
		IR	191.29	0.15	0.08	4.17		
	C	Red	475.80	–	–	–	–	99
		IR	188.14	–	–	–		
	D	Red	408.78	0.52	0.13	5.98	99.5	99
		IR	178.12	0.10	0.06	4.29		
10	A	Red	723.36	0.20	0.03	9.23	–	99
		IR	188.22	–	–	–		
	B	Red	389.73	0.26	0.07	8.84	–	97
		IR	199.85	–	–	–		
	C	Red	401.31	0.51	0.13	8.30	–	98.1
		IR	199.34	–	–	–		
	D	Red	385.26	0.24	0.06	6.86	97.3	97.5
		IR	182.33	0.1	0.05	6.27		
